# Physiological changes after fluid bolus therapy in cardiac surgery patients: A propensity score matched case–control study

**DOI:** 10.1016/j.ccrj.2023.11.005

**Published:** 2024-01-12

**Authors:** Martin Faltys, Ary Serpa Neto, Luca Cioccari

**Affiliations:** a Department of Intensive Care Medicine, Inselspital, Bern University Hospital, University of Bern, Bern, Switzerland; b Department of Intensive Care, Austin Hospital, Melbourne, Australia; c Department of Critical Care Medicine, Hospital Israelita Albert Einstein, São Paulo, Brazil; d Australian and New Zealand Intensive Care Research Centre, School of Public Health and Preventive Medicine, Monash University, Prahran, VIC 3004, Australia; e Department of Critical Care, The University of Melbourne, Melbourne, Australia; f Data Analytics Research and Evaluation Centre, Austin Hospital, Melbourne, Australia; g Department of Intensive Care Medicine, Kantonsspital Aarau, Aarau, Switzerland

**Keywords:** Cardiac surgery, Resuscitation, Intensive care, Hypotension

## Abstract

**Objective:**

Fluid bolus therapy (FBT) is ubiquitous in intensive care units (ICUs) after cardiac surgery. However, its physiological effects remain unclear.

**Design:**

**:** We performed an electronic health record–based quasi-experimental ICU study after cardiac surgery. We applied propensity score matching and compared the physiological changes after FBT episodes to matched control episodes where despite equivalent physiology no fluid bolus was given.

**Setting:**

The study was conducted in a multidisciplinary ICU of a tertiary-level academic hospital.

**Participants:**

The study included 2,736 patients who underwent Coronary Artery Bypass Grafting and/or heart valve surgery.

**Main Outcome Measures:**

Changes in cardiac output (CO) and mean arterial pressure (MAP) during the 60 minutes following FBT.

**Results:**

We analysed 3572 matched fluid bolus (FB) episodes. After FBT, but not in control episodes, CO increased within 10 min, with a maximum increase of 0.2 l/min (95%CI 0.1 to 0.2) or 4% above baseline at 40 min (p < 0.0001 vs. controls). CO increased by > 10% from baseline in 60.6% of FBT and 49.1% of control episodes (p < 0.0001). MAP increased by > 10% in 51.7% of FB episodes compared to 53.4% of controls. Finally, FBT was not associated with changes in acid-base status or oxygen delivery.

**Conclusion:**

In this quasi-experimental comparative ICU study in cardiac surgery patients, FBT was associated with statistically significant but numerically small increases in CO. Nearly half of FBT failed to induce a positive CO or MAP response.

## 1 Introduction

Fluid bolus therapy (FBT) is common in patients admitted to the intensive care unit (ICU) after cardiac surgery. It is prescribed based on the assumption that it will increase effective circulating volume and thereby, cardiac index, mean arterial pressure, oxygen delivery, and urinary output. However, the nature, extent, and duration of the physiological responses to FBT have been questioned.[1], [2], [3]

There is no consensus definition of FBT. In an international survey of ICU specialists, the most frequently accepted definition of FBT was 250 mL of either an intravenous colloid or crystalloid given over less than 30 min.^4^ From a physiological perspective, this appears reasonable, as the proportion of fluid responders decreases when the infusion time exceeds 30 min.^5^ A positive response to a fluid bolus (FB) is commonly defined as an increase in cardiac output (CO) by > 10–15%. However, many clinicians also consider an increase in mean arterial pressure (MAP), urinary output, central venous oxygen saturation, or a decrease in heart rate (HR) or blood lactate levels to be a positive response.^4^ Notably, there is no consensus on what defines a positive response for these variables either.

Despite its common use, detailed data on the physiological effects of FBT in ICU patients are scarce, lack statistical robustness, and are limited to the period immediately following an FB, even when dynamic indices of likely responsiveness such a stroke volume or pulse pressure variation or the leg raising test are present.^2^ Thus, the response to FBT is frequently assessed within only the first 10 min after completion.^5^ When the observation period is extended, the effect appears to dissipate within minutes.^6^^,^^7^ On the other hand, FBT contributes to the positive fluid balance often seen in patients after cardiac surgery,^8^ which has been associated with increased mortality and higher hospital costs.^9^

The above considerations imply the need to obtain more robust data on the physiological effects of FBT beyond the period immediately after its administration. Accordingly, we sought to evaluate the physiological changes associated with FBT in patients after cardiac surgery using a large and detailed dataset from the electronic health records (EHR) of a tertiary academic ICU.

## 2 Materials and methods

The research ethics committee of the canton of Bern (Kantonale Ethikkommission Bern, KEK) approved the study (KEK approval number 2016-01463) and waived the need for written informed consent.

We studied all adult patients (≥18 years) admitted to the ICU after coronary artery bypass grafting (CABG) and/or heart valve surgery at the University Hospital of Bern, Switzerland, from January 2008 to July 2019. We excluded patients without documented invasive blood pressure measurements, patients treated with a circulatory assist device (including ventricular assist devices, extracorporeal membrane oxygenation, and impeller-based devices), or patients who received protocolized fluid therapy in the setting of a clinical trial. We obtained the complete ICU EHR dataset of the included patients. Continuously measured physiological variables (hemodynamic data, infusion pump rates) are stored every 2 min in the electronic patient data management system (PDMS; GE Centricity Critical Care, General Electrics, Helsinki, Finland) as part of routine care. This includes the pulmonary artery catheter (PAC) measurements, mixed venous oxygen saturation (SvO2), pulmonary artery pressure (PAP), and in particular, Stroke volume (SV) and CO measurements (Swan-Ganz pulmonary artery catheter with continuous thermodilution, Edwards Lifesciences, Irvine, CA). The pulmonary capillary wedge pressure (PCWP) was recorded as performed by the clinician. All interventions (e. g., fluid therapy) are recorded with time stamps at the start and end of the intervention. Fluid administration is recorded by the bedside nurses immediately after administration with the timestamp matching the actual administration time. Institutional practice puts great importance on the accuracy of these recordings.

### 2.1 Fluid bolus and observation period

Ringer's lactate (RL) was the only crystalloid solution administered as an FB in cardiac surgery patients according to our institutional protocol. We defined an FB as the continuous administration of ≥250 ml RL within ≤30 min (corresponding to an infusion rate >500 ml/h). For each FB, an observation period was defined as ranging from 60 min before the start to 60 min after the end of the FB. A detailed description of the fluid bolus definition is included in the appendix (Additional file 1: Figure e1). Episodes of FBT were considered for analysis only if they were isolated, e.g. if the patient did not receive any other FB during the entire observation period. The translated excerpt of the institution's hemodynamic protocol is included in Additional file 1: Figure e2.

### 2.2 Propensity score matching

We compared the physiological changes after FBT with changes occurring in so-called control episodes (time epochs of equal duration, preceded by similar hemodynamic baseline characteristics, and similar time since ICU admission, but without fluid administration).

For every fluid bolus given, we randomly sampled two control episodes from the same patient where no fluid was administered. The duration of observation for each control episode was identical to the observation period described above.

We used one-to-one propensity score matching (PSM) without replacement to pair FBT episodes and control episodes with similar baseline hemodynamic characteristics and covariates (Additional file 1: Method Supplement). The *matched* fluid bolus and control episodes, however, did not have to be from the same patient. We specify all details of our PSM approach in Additional file 1: Method Supplement

### 2.3 Outcomes

The main objective of this study was to evaluate the hemodynamic changes associated with FBT, namely the changes in CO, SV, HR, MAP, vasoactive-inotropic score (VIS), Systemic Vascular Resistance (SVR), SBP, diastolic blood pressure (DBP), central venous pressure (CVP), PAPm, PCWP, and perfusion pressure (MAP – CVP) from baseline until 60 min after the fluid bolus. The VIS^10^ expands the previously described inotropic score^11^ and combines the administered vasoactive and inotropic drugs into one score indicative of pharmaceutical cardiovascular support. Therefore, higher VIS scores imply a higher dose of vasoactive or inotropic support.

Secondary outcomes included changes in metabolic and oxygen delivery variables and an analysis of the effect of FB volume and infusion rate on CO, SV, and MAP. We considered the following oxygen delivery and metabolic variables: arterial oxygen saturation (SaO2), hemoglobin (Hb), oxygen delivery (DO2), SvO2, blood lactate levels, pH, base excess (BE), and urine output.

### 2.4 Statistical analysis

We compared baseline values before the FB to values in the 60 min after the end of the FB against the changes observed in the matched control episodes using linear mixed-effects models (LMM)^12^ to account for repeated measurements. We used the binary indicator of FB, time after FB and the baseline value as fixed effects and patients as random effect and report the P-value for the FB indicator and for its interaction with time after FB. We report mean values with 95% confidence intervals at all time-points. We calculated three separate linear regression models to estimate the median effect of bolus size and infusion rate on MAP, CO, and SV. Besides bolus size and rate, the models include the baseline values of HR, MAP, SBP, DBP, CVP, VIS, increase in VIS from baseline, patient age, gender, and height. For the CO and SV model, additional predictors were baseline CO and SV. The ratio of FB responders (defined as >10% increase in the variable of interest) in the FB and control episodes was compared using Pearson's chi-squared test. Bonferroni correction was applied to account for multiple testing. With 31 tests of interest, choosing an overall significance level of 0.05 resulted in α = 0.0016 for statistical significance for the individual tests.

All data preprocessing was done using Python 3.8.^13^ R^14^ was used for the statistical analysis, including propensity score matching with the library matchit^15^ and the linear mixed-effect models using lmerTest.^16^ The code and all used libraries can be found on https://github.com/mafacz/2020_FBT.

## 3 Results

### 3.1 Patient and bolus characteristics

**Fig. 1:**
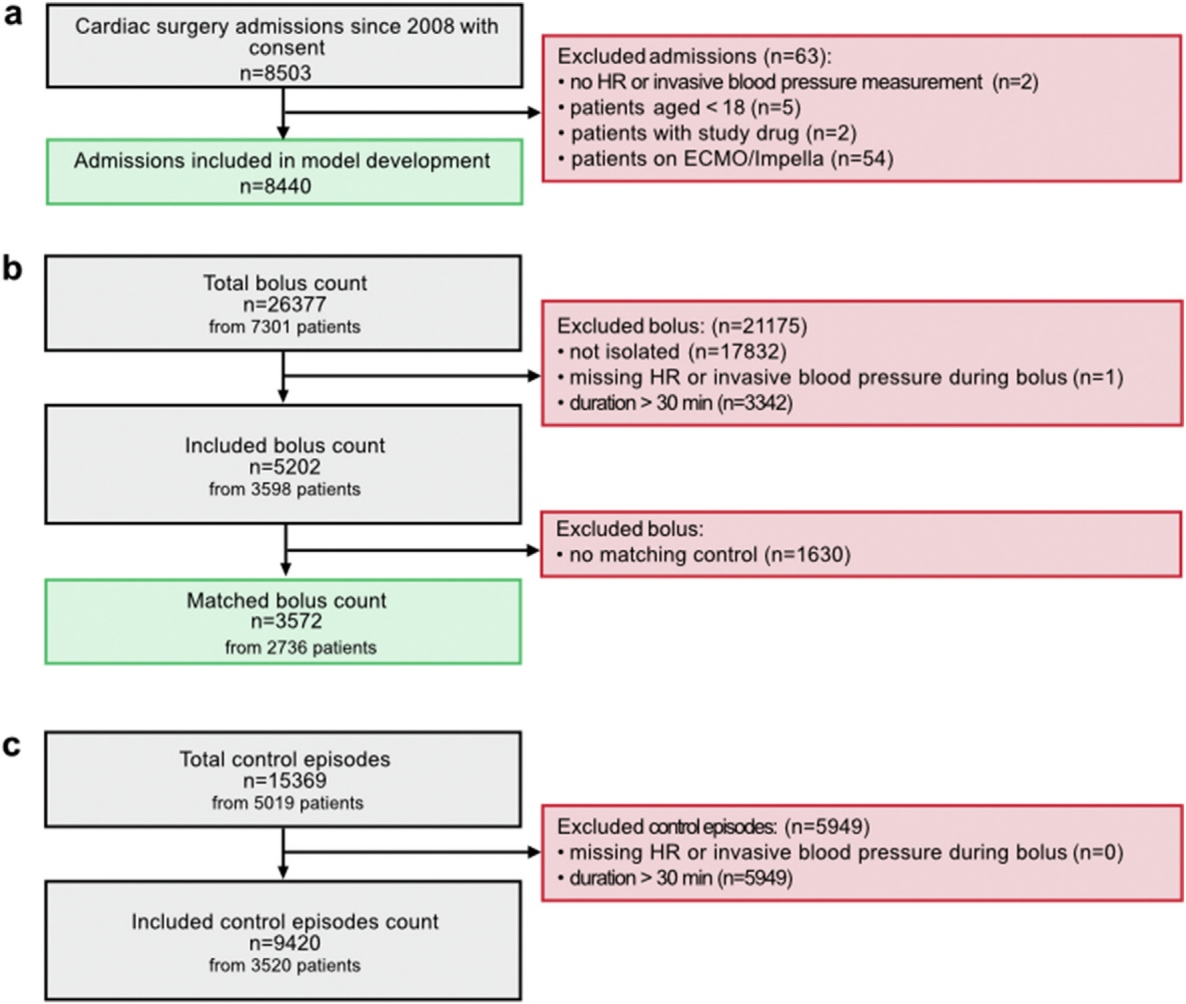
**Patient and FBT flow chart.** **a)** Patient's admissions with reasons for exclusion. **b)** FB counts with reasons for exclusion and effects of propensity score matching on numbers included. **c)** Control episodes counts with reasons for exclusion.

We identified 8503 patients after cardiac surgery during the study period. After excluding 63 patients who met our exclusion criteria, we considered 8440 patients (Fig. 1a). There were 26,377 FBs administered to 7301 patients, of which 5202 were isolated FBs in 3598 patients (Fig. 1b). Using the same approach, we included 9420 control episodes (Fig. 1c). Patients’ demographics and baseline characteristics are shown in Table 1. The median observed FB size was 355 ml (IQR 186 ml) administered over a mean of 18.2 min (SD 9.0). Details on bolus size and duration are provided in Additional file 1: Figure e3.

**Table 1:** Patient characteristics.

	Overall	Matched	
		Control	Bolus
**N**	8440	3572	3572
**Female (%)**	2176 (25.8)	995 (27.9)	985 (27.6)
**Age at admission (years)**	66.93 (11.45)	68.58 (11.04)	68.40 (11.03)
**Height (cm)**	170.89 (20.18)	169.97 (9.88)	170.31 (9.83)
**Weight (kg)**	80.35 (17.67)	77.86 (15.66)	78.28 (15.78)
**APACHE II score**	17.39 (6.23)	18.50 (6.44)	18.39 (6.46)
**Admission type emergency (%)**	486 (5.8)	253 (7.1)	253 (7.1)
**Cardiac surgery types (%)**			
CABG	3723 (44.1)	1619 (45.3)	1537 (43.0)
Heart valve surgery	3087 (36.6)	1078 (30.2)	1192 (33.4)
CABG + Heart valve surgery	1630 (19.3)	875 (24.5)	843 (23.6)
**ICU length of stay (days)**	1.22 (1.82)	1.60 (2.68)	1.96 (3.70)
**Mechanical ventilation (%)**	4621 (54.8)	2790 (78.1)	2816 (78.8)
**Time from ICU admission to fluid bolus (h)**		10.6 [9.5]	7.9 [8.8]
**ICU mortality (%)**	52 (0.6)	67 (1.9)	60 (1.7)

Values are presented as mean (SD: standard deviation), median [Interquartile Range] or numbers (%). APACHE: Acute Physiology And Chronic Health Evaluation. CABG: Coronary Artery Bypass Grafting. ICU: Intensive Care Unit.

**Fig. 2:**
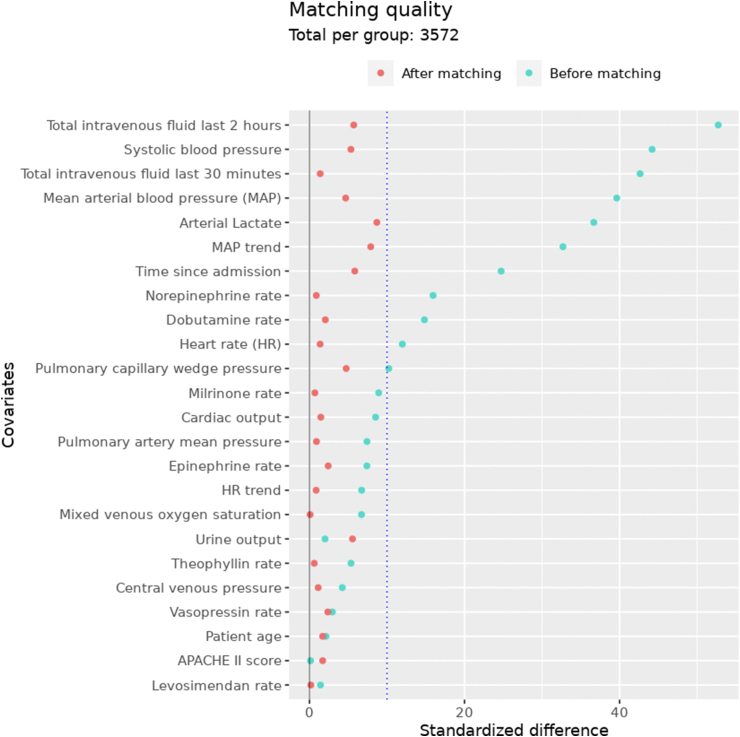
**Propensity score matching quality.** The average absolute standardized difference (SDiff) of all non-binary covariates considered in propensity score matching (PSM). Comparing the values before and after PSM. An SDiff <10% is commonly regarded as good matching quality.

**Fig. 3:**
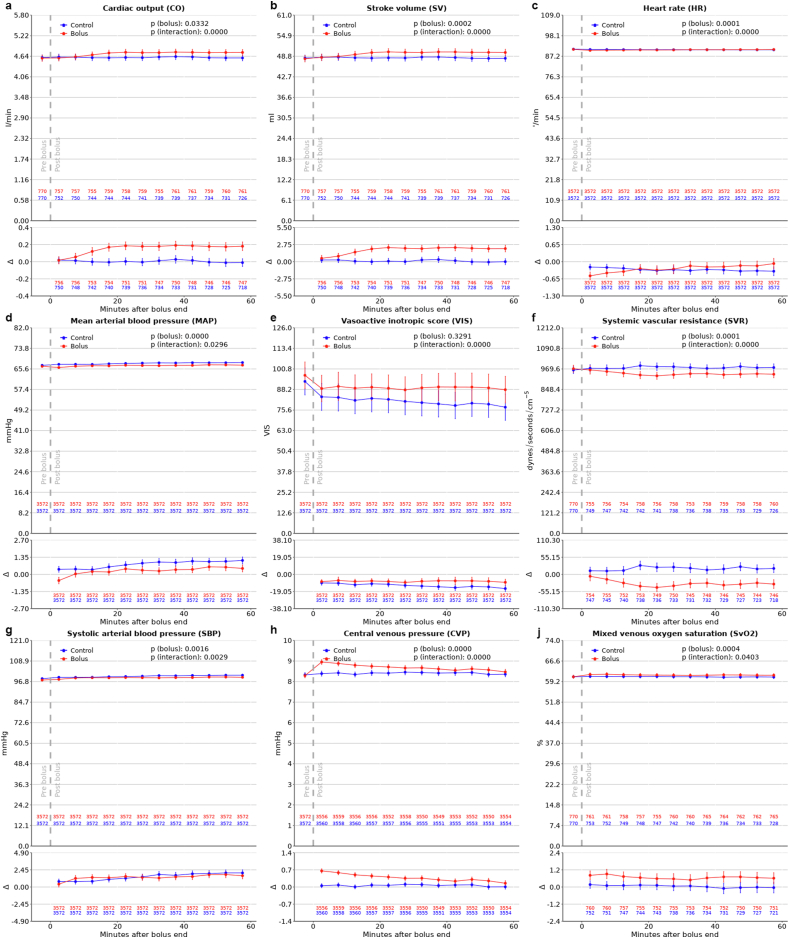
**Hemodynamic effects of FBT**. a–j: For each variable, the top plot shows the average value of 30 min before the fluid bolus therapy (before gray line) and the average values of 5 min intervals from 0 to 60 min after the FB end. In the lower plot the values are the mean of the changes from baseline calculated for each time point individually. In both plots, this is shown for the FBT and control episodes separately. The 95% confidence intervals are shown as error bars. The red and blue numbers represent the number of measurements at each time point for FB (red) and control episodes (blue).

We obtained 3572 matched pairs of FB and control episodes from 2736 patients using PSM. Of these patients, 1770 (65%) had both a fluid bolus and control episode selected. All covariates had a standardized difference below 10% after matching (Fig. 2, additional file 2: Table e1). Table 2 and Additional file 2: Table e2 list the baseline values of all key variables for FBT and control episodes. The number of observations from the PAC decreased over the observation period of 1 h after the respective FBT indicating that some PAC were removed during this period (<6%, see Fig. 3).

**Table 2:** Additional hemodynamic, metabolic, and oxygen delivery outcomes.

			Baseline	Observation Period											
				5 min	10 min	15 min	20 min	25 min	30 min	35 min	40 min	45 min	50 min	55 min	60 min
**Perfusion pressure**	p (bolus): <0.0001 p (interaction): 0.8133	Bolus	58.35 (58.03–58.67)	57.21 (56.86–57.56)	57.79 (57.44–58.14)	58.06 (57.70–58.43)	58.06 (57.72–58.41)	58.36 (58.01–58.72)	58.30 (57.95–58.64)	58.21 (57.88–58.55)	58.37 (58.03–58.71)	58.46 (58.12–58.80)	58.60 (58.25–58.94)	58.64 (58.30–58.99)	58.63 (58.29–58.97)
		Control	58.71 (58.40–59.02)	59.05 (58.72–59.38)	59.04 (58.71–59.37)	59.06 (58.73–59.40)	59.21 (58.88–59.54)	59.39 (59.05–59.72)	59.47 (59.12–59.83)	59.59 (59.25–59.93)	59.57 (59.23–59.91)	59.65 (59.30–59.99)	59.61 (59.26–59.95)	59.70 (59.36–60.04)	59.77 (59.43–60.12)
**DBP**	p (bolus): <0.0001 p (interaction): 0.4348	Bolus	51.49 (51.24–51.74)	50.61 (50.35–50.86)	51.06 (50.79–51.32)	51.25 (50.97–51.52)	51.25 (50.99–51.52)	51.47 (51.20–51.74)	51.42 (51.16–51.69)	51.32 (51.06–51.57)	51.45 (51.19–51.71)	51.53 (51.27–51.79)	51.60 (51.33–51.86)	51.58 (51.31–51.84)	51.59 (51.32–51.86)
		Control	51.65 (51.41–51.88)	51.89 (51.63–52.14)	51.87 (51.61–52.12)	51.85 (51.60–52.11)	52.01 (51.75–52.27)	52.13 (51.86–52.39)	52.16 (51.89–52.43)	52.14 (51.88–52.40)	52.16 (51.89–52.42)	52.23 (51.96–52.49)	52.18 (51.91–52.45)	52.17 (51.90–52.44)	52.22 (51.96–52.49)
**PAPm**	p (bolus): 0.0047 p (interaction): 0.0504	Bolus	22.97 (22.58–23.37)	23.76 (23.35–24.16)	23.66 (23.26–24.06)	23.65 (23.25–24.05)	23.55 (23.13–23.97)	23.58 (23.17–23.98)	23.61 (23.20–24.02)	23.53 (23.13–23.94)	23.37 (22.97–23.78)	23.32 (22.92–23.72)	23.36 (22.95–23.77)	23.43 (23.01–23.85)	23.50 (23.06–23.95)
		Control	23.02 (22.64–23.41)	23.38 (22.97–23.80)	23.38 (22.96–23.81)	23.44 (23.02–23.86)	23.58 (23.14–24.01)	23.61 (23.15–24.06)	23.46 (23.04–23.89)	23.28 (22.86–23.71)	23.51 (23.05–23.97)	23.47 (23.04–23.89)	23.41 (22.98–23.84)	23.20 (22.78–23.62)	23.37 (22.93–23.80)
**SpO2**	p (bolus): <0.0001 p (interaction): <0.0001	Bolus	97.75 (97.68–97.83)	97.88 (97.80–97.96)	97.84 (97.75–97.92)	97.79 (97.70–97.87)	97.78 (97.70–97.86)	97.71 (97.63–97.80)	97.70 (97.62–97.78)	97.70 (97.61–97.78)	97.62 (97.54–97.71)	97.62 (97.53–97.70)	97.63 (97.54–97.71)	97.55 (97.46–97.64)	97.56 (97.47–97.64)
		Control	97.48 (97.41–97.56)	97.48 (97.40–97.56)	97.39 (97.31–97.48)	97.35 (97.26–97.44)	97.39 (97.31–97.48)	97.40 (97.32–97.48)	97.36 (97.27–97.44)	97.34 (97.26–97.43)	97.34 (97.26–97.43)	97.36 (97.27–97.44)	97.35 (97.27–97.43)	97.31 (97.23–97.40)	97.28 (97.20–97.37)

	**FBT**		**Control**					**FBT**		**Control**					
	*Baseline*	*Observation* *Period*	*Baseline*	*Observation* *Period*				*Baseline*	*Observation* *Period*	*Baseline*	*Observation* *Period*				

**PCWP** (p = 0.2607)	13.62 (13.13–14.11)	14.19 (13.70–14.69)	13.77 (13.24–14.31)	13.53 (13.02–14.04)			**ScvO2** (p = 0.1751)	59.05 (57.88–60.21)	59.38 (58.40–60.35)	59.60 (58.42–60.78)	59.62 (58.41–60.82)				
**Urine** (p = 0.0465)	98.49 (94.59–102.39)	87.88 (84.23–91.52)	93.56 (89.54–97.58)	81.56 (78.33–84.80)			**pH** (p = 0.7084)	7.42 (7.42–7.43)	7.44 (7.43–7.44)	7.43 (7.43–7.43)	7.43 (7.43–7.44)				
**Lactate** (p = 0.0075)	1.99 (1.90–2.07)	2.11 (2.00–2.22)	1.88 (1.79–1.97)	1.73 (1.65–1.81)			**paO2** (p = 0.2190)	111.39 (108.15–114.62)	103.15 (100.98–105.33)	106.87 (104.37–109.36)	102.10 (99.85–104.34)				
**BE** (p = 0.5063)	−0.73 (−0.90–0.55)	−0.62 (−0.80–0.44)	−0.30 (−0.47–0.13)	−0.32 (−0.48–0.16)			**SaO2** (p = 0.3993)	97.18 (96.85–97.51)	97.39 (97.18–97.60)	97.45 (97.25–97.65)	97.44 (97.29–97.59)				
**Hb** (p < 0.0001)	101.3 (100.2–102.4)	97.2 (96.1–98.3)	99.2 (98.1–100.3)	101.1 (100.00–102.2)			**dO2** (p = 0.1222)	571.79 (543.97–599.61)	577.11 (554.58–599.64)	583.45 (557.16–609.74)	614.72 (591.05–638.39)				

Reported in 5-min intervals for continuously measured variables and as one value for sporadically measured variables (Values are presented as mean (95% confidence interval)). P-value for intergroup difference. DBP: Diastolic blood pressure, PAPm: mean pulmonary arterial pressure, SpO2: peripheral capillary oxygen saturation, PCWP: pulmonary capillary wedge pressure, BE: base excess, Hb: Hemoglobin, ScvO2: Central venous oxygen saturation, paO2: arterial oxygen partial pressure, SaO2: arterial oxygen saturation, dO2: Global oxygen delivery, FBT: Fluid bolus therapy.

### 3.2 Cardiovascular effects

After FBT, CO increased within 10 min, for a maximum increase by 0.2 l/min (95%CI 0.1 to 0.2) or 4 % above baseline after 40 min (Fig. 3a). CO remained unchanged in controls (P-value for the difference between groups <0.0001).

Stroke volume increased by 4.2% to a maximum of 2.3 ml (95%CI 1.78 to 2.77) above baseline 25 min after FBT, while it remained unchanged in controls (Fig. 3b) (p < 0.0001). HR changes were negligible (Fig. 3c); however most (71.2%) patients were epicardially paced. Beta blockers were used during less than 0.2% of episodes.

In the first 5 min after an FB, MAP decreased by 0.5 mmHg (95%CI 0.2 to 0.7) and returned to baseline after 10 min. The maximum MAP increase after FBT was 0.6 mmHg (0.3–0.8) above baseline after 50 min. In the control group, MAP increased by 1.1 mmHg (95%CI 0.8 to 1.4) above baseline after 1 h (p < 0.0001) (Fig. 3d). Moreover, while the VIS decreased by a maximum of 9.0 units (95%CI 6.3 to 11.6) in the FBT group, it decreased by 15.8 units (95%CI 12.5 to 19.1) for control episodes (Fig. 3e) (p < 0.0001). Coherent with these changes, SVR decreased in the FBT group and increased in controls (Fig. 3f).

The increase in SBP by 1.8 mmHg (95%CI 1.3–2.2) (Fig. 3g) and CVP by 0.7 mmHg (95%CI 0.6–0.7) was small (Fig. 3h). Additional file 2: Table e1 lists the hemodynamic values including results for all vasoactive/inotropic drugs and detailed P-values. Coherent with changes in MAP and CVP, perfusion pressure decreased by 1.1 mmHg (95%CI 0.9 to 1.4) and returned to baseline 25 min after FBT, whereas, for controls, it increased by 1.1 mmHg (95%CI 0.8 to 1.4) (p < 0.0001) (Table 2). We observed minor changes in DBP, PCWP, and PAPm (Table 2).

In the regression analysis, MAP decreased by 0.3 mmHg (95%CI 0.2 to 0.3, p < 0.001) for each 100 ml of fluid received, while CO increased by 22.4 ml/min (95%CI 5.6 to 39.1) and SV increased by 0.3 ml (95%CI 0.1 to 0.5) per 100 ml received. The results for changes in CO and SV were not statistically significant after correction for multiple comparisons. FBT infusion rate had no observed effect in the models. Model details are shown in Additional file 2: Table e3. We present the changes after the most rapidly infused FBs (top tercile, median rate 50 ml/min, IQR 33 ml/min) and for only the first FBT of a patient as a sensitivity analysis separately in Additional file 1: Figure e4 and Figure e5 respectively.

### 3.3 Fluid responders vs. non-responders

CO increased by > 10% from baseline in 60.6% of FB and 49.1% of control cases (p < 0.0001). In responders, CO increased within the first 20 min and then remained elevated. SV increased by >10% in 62.3% of FB and 50.5% of control cases (p < 0.0001).

MAP increased by > 10% in 51.7% of FB cases (with a simultaneous VIS increase by > 10% in 10.6%), compared to 53.4% of the control cases (simultaneous VIS increase >10% in 6.1%). The visualization of the change over time for the three variables is in Additional file 1: Figure e6. The difference in MAP increase between the groups was not significant (p = 0.1484). Finally, VIS increased by > 10% in 8.9% of all FB cases vs. 5.1% of controls (p < 0.0001) and decreased by >10% in 8.4% of all FB cases vs. 8.1% of controls (p = 0.6990).

### 3.4 Secondary outcomes

Compared to controls, in the FBT group, we observed statistically significant overall differences in SpO2, SvO2, and Hb, but not in DO2, ScvO2, and PaO2 (Table 2). However, the peak changes in SpO2 and SvO2 were clinically negligible (Fig. 3j). The hemoglobin concentration decreased by 0.4 g/dl (CI95% 0.2–0.6) after FBT, while it increased by 0.9 g/dl (CI95% 0.5–1.3) in controls (Table 2). This did not result in a statistically significant change in systemic oxygen delivery, but we observed a numerical decrease in DO2 by 14.2 ml/min (CI95% −72.8 to 44.3) after FBT and a numerical increase by 41.9 ml/min (CI95% −10.2 to 94.0) in control episodes (Table 2). We did not observe any statistically significant effects on urine output, pH, BE, or lactate (Table 2).

## 4 Discussion

### 4.1 Key findings

In this propensity score–matched analysis of 3572 isolated FBT administered after cardiac surgery, we found statistically significant but numerically small changes in CO, MAP, HR, CVP, SBP, DBP, SV, SVR, SvO2, perfusion pressure, and vasoactive drug use over 60 min of observation. Overall, CO increased by a maximum of 0.2 l/min after FBT and a positive fluid response (increase in CO by > 10% from baseline) was observed in 60.6% of FB episodes. CO remained unchanged in controls. Similarly, an increase in MAP by > 10% from baseline was observed in only 52% of FB episodes compared to 53 % of the control cases. In the FBT group, we also observed a statistically significant decrease in Hb concentration, without statistically significant changes in oxygen delivery, urine output, or blood lactate levels.

### 4.2 Relationship to previous literature

The majority of studies of FBT in critically ill patients have assessed the response to a fluid bolus at the end of the infusion or within few minutes thereafter.^2^^,^^6^^,^^17^ In those studies looking beyond the time immediately after FBT, hemodynamic data were obtained intermittently rather than continuously, and the observed hemodynamic changes decreased gradually over time.^18^^,^^19^ Comparing the effects of 500 ml warm (40 °C) versus room-temperature crystalloids bolus infusion in 50 cardiac surgery patients, the percentage of patients with an increase of CI by > 15% was similar in both groups and in the order of 60% (64% vs. 60%).^20^ In 20 mostly septic-shocked ICU patients, 500 mL of crystalloids over 30 min increased CI in 13 (65%);^19^ however, this effect dissipated within 60 min. In another study^6^ of 26 postoperative critically ill patients, 250 mL of crystalloid over 5 min increased CI in 13 patients (50%), with dissipation within 10 min. In 57 children receiving FBT after cardiac surgery, the MAP was unpredictable, with a poor relationship between CO and MAP responsiveness^21^ and low response rates similar to adults.^22^ Our findings are aligned with and expand the above prospective investigations. Unlike our study, no previous studies have presented a hemodynamically equivalent control population who did not receive FBT.

In the postoperative period, the hemodynamic effect half-life of crystalloid FBT appears short.^23^ Proposed mechanisms include relaxation of venous vessels, redistribution of fluid to the compliant venous plexuses, capillary leakage, and reduction in smooth vascular tone through sympathetic inhibition.^6^ Previous research in healthy volunteers^24^ has shown that fluid temperature may also affect hemodynamic responses.^25^^,^^26^ Finally, our observations that SVR decreases with FBT are aligned with observations in volunteers that showed the same effects. This is likely secondary to a reduction in viscosity.^27^

### 4.3 Implications of study findings

Our findings support the results of smaller clinical studies.[28], [29], [30], [31], [32] First and foremost, almost 40% of the >3500 FB episodes analyzed in this study failed to increase CO by > 10%, and almost 50% of FB episodes failed to increase MAP, suggesting that in clinical practice, administration of ineffective FBT may be common. Furthermore, our findings suggest that, when present, the physiological effects of an isolated crystalloid fluid bolus may be small. Therefore, our results emphasize the need for a comprehensive assessment of an individual patient's likelihood to benefit from FBT^33^ before administering a fluid bolus.^34^ This is all the more important because the expectations of intensive care physicians regarding the physiological effects of FBT are often inaccurate.^35^ Several studies demonstrated a correlation between high VIS and poor outcome. The VIS can be used to objectively quantify the degree of hemodynamic support. However, the VIS has some pitfalls, including the fact that a universally recognized version that includes all commonly used vasoactive drugs does not exist.^36^ The VIS incorporates effects from agents with vasoconstrictor, vasodilator and inotropic properties or combinations thereof. The main goal of FBT at the bedside is to optimize cardiac filling, in order to increase SV and CO. If such hemodynamic improvement is obtained, the VIS is expected to decrease. Much to our surprise, no such effect was detectable in our cohort. Instead, we observed a significant increase in VIS in 8.9% of all FB cases.

### 4.4 Strengths and limitations

To the best of our knowledge, this is the largest analysis of FBT effects in patients after cardiac surgery published to date. We used propensity score matching to identify hemodynamically indistinguishable control periods to create a quasi-experimental, digital, controlled trial-like comparison. We obtained granular data from continuous automatic electronic recordings of hemodynamic parameters and analyzed >3500 matched fluid boluses in >2700 patients. By considering only isolated fluid boluses and accounting for the confounding effect of vasopressors and inotropes, we were able to comprehensively explore the physiological effects of FBT up to 1 h after the fluid bolus (longer than all previous cardiac surgery FBT studies in the literature).

The results of this study should be interpreted with caution and considered exploratory. Obviously, the single-center retrospective design comes with inherent limitations. However, to our knowledge there are no large detailed multi-center data sets suitable to address our research question. Propensity score matching has some limitations as it relies on the assumption that all confounding variables are included. Imperfect matches result in discarded data, reducing the effective sample size, and the results depend on appropriate choice of matching algorithm and specific caliper. However, with large, granular datasets, propensity score matching is a valuable approach for controlling confounding variables and examining causal relationships if randomized controlled trials are not available or feasible. We do not know the clinical indications for the fluid administered to our patients and we cannot make assumptions regarding their fluid status^37^ at the time of FBT. However, all patients were assessed by the treating physician and treated according to the institutional hemodynamic protocol, which required clinicians to consider fluid administration for low blood pressure, prolonged capillary refill time >3 s, and low cardiac output. Therefore, we can reasonably assume that a physician assessing the patient regarded the FB as indicated and likely beneficial to the patient's hemodynamics before administration. By excluding repeated FB episodes and analyzing only isolated FB, we might have introduced selection bias to the extent that our cohort may not represent the most hemodynamically unstable patients. However, in this context, our finding that 40%–50% of the >3500 FB given to >2700 patients failed to improve CO and MAP is even more relevant. Importantly such FBs contribute to the positive fluid balance seen in patients after cardiac surgery,^8^ which has been associated with harm.^9^ As the only type of fluid used in our ICU is Ringer's lactate, we cannot make inferences on the physiological effects of other types of fluid, e.g. colloids. However, balanced crystalloids are the preferred fluid type in post-cardiac surgery patients,^38^ and studies assessing the effect of 20% albumin for FBT^7^^,^^26^^,^^39^ reported findings similar to ours.^7^^,^^40^ The fluid boluses may have been too small to produce an effect. However, our definition is in accordance with previous literature,^4^ a quarter of our patients (>1000) received more than 500 ml of fluids, and fluid bolus size was not an independent predictor of response. Similarly, FBT infusion rate had no observed effect in the models. Finally, some may argue that, if dynamic tests of likely fluid responsiveness (stroke volume or pulse pressure variation or leg raising) had been systematically applied, the percentage and magnitude of responders would have been greater. However, such tests have never assessed hemodynamics for the hour following FBT, have never been compared with controls, and have not shown predictive superiority in cardiac surgery patients.^41^

Our findings must not be interpreted to indicate that FBT has no effect in critically ill patients. In patients undergoing major surgery, and those with hypovolemia due to trauma or sepsis, adequate fluid administration is essential for the maintenance of tissue perfusion. The degree of intravascular volume expansion is context-sensitive.^42^^,^^43^ However, patients after cardiac surgery present with distinct pathophysiological features that are very different from the general ICU population. These include a positive fluid balance from the pump prime and cardioplegia solution, the frequent use of vasoactive drugs, the presence of systolic and/or diastolic dysfunction in many patients, and the need for epicardial pacing in most cases. Accordingly, assessment of the effects of FBT in these patients cannot be based on extrapolated data from other conditions where FBT is also administered.^44^

## 5 Conclusions

We conducted a quasi-experimental, digital, controlled trial-like comparison and propensity score–matched analysis of >3500 isolated FBT episodes after cardiac surgery. Compared with matched control episodes, FBT episodes were associated with statistically significant but numerically small increases in CO, CVP, and SV. A significant proportion (40%–50%) of FB episodes failed to induce a positive CO or MAP response, indicating that a substantial amount of fluid administered after cardiac surgery does not improve hemodynamics. These findings suggest the need to conduct randomized controlled studies to more clearly define the role of FBT vs. vasoactive drug adjustments in cardiac surgery patients admitted to the ICU. In the meantime, clinicians should bear in mind the importance of carefully evaluating the need for a fluid bolus in these patients.

## Funding

This research did not receive any specific grant from funding agencies in the public, commercial, or not-for-profit sectors.

## Authors’ contributions

All authors contributed to the study’s conception and design. MF performed data extraction. Data Analysis was performed by MF and ASN under the guidance of LC. MF and LC wrote the first draft of the manuscript and all authors revised the manuscript for important intellectual content. All authors read and approved the final manuscript.

## Availability of data and materials

The datasets used and/or analyzed during the current study are available from the corresponding author on reasonable request.

## Conflict of interest

The authors declare that they have no known competing financial interests or personal relationships that could have appeared to influence the work reported in this paper.
