# Supplementary material for: Physiological changes after fluid bolus therapy in cardiac surgery patients: A propensity score matched case–control study
Source: Crit Care Resusc. 2024 Jan 12;26(1):32–40. doi: 10.1016/j.ccrj.2023.11.005 (PMC11056405; doi:10.1016/j.ccrj.2023.11.005)
Supplement: Multimedia component 1 [file mmc1.docx]

FBT in cardiac surgery – Online Figures and Resources

**Physiological changes after fluid bolus therapy in cardiac surgery patients: a propensity score matched case-control study**

Martin Faltys ^1,2^ (ORCID 0000-0001-5579-810X), Ary Serpa Neto ^3,4,5,6^, Luca Cioccari ^1,7^

1 Department of Intensive Care Medicine, Inselspital, Bern University Hospital, University of Bern, Bern, Switzerland

2 Department of Intensive Care, Austin Hospital, Melbourne, Australia

3 Department of Critical Care Medicine, Hospital Israelita Albert Einstein, São Paulo, Brazil

4 Australian and New Zealand Intensive Care Research Centre, School of Public Health and Preventive Medicine, Monash University, Prahran, VIC 3004, Australia

5 Department of Critical Care. The University of Melbourne, Melbourne, Australia

6 Data Analytics Research and Evaluation Centre, Austin Hospital, Melbourne, Australia

7 Department of Intensive Care Medicine, Kantonsspital Aarau, Aarau, Switzerland

**Fig. e1**:


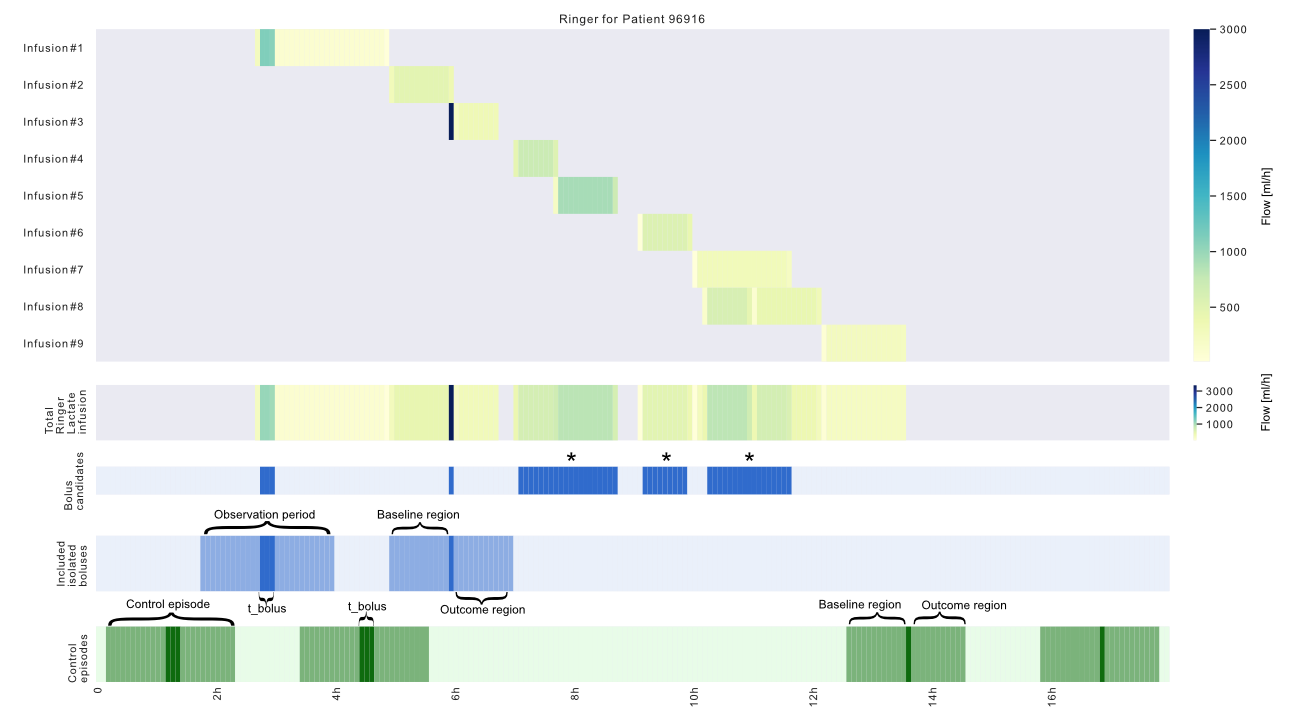


**Fig e1 Exemplary ICU patient stay**.

This patient received nine infusions during his stay (topmost plot). We derived the administered total fluid flow at any time point by summation. The fluid bolus (FB) candidates satisfy our bolus definition but might have other FB during the observation period (such as the ones marked with an Asterix). We considered only isolated FB for the purpose of this study, the first two in this case. Every FB has a baseline region before and an outcome region after the bolus. The duration of the FB itself is t_bolus_. For each included bolus we sampled two control episodes (green) without FB administration during the whole observation period. The baseline and outcome regions of the control are separated by t_bolus_ of the index FB. We defined a fluid bolus as the continuous administration of ≥ 250 ml RL within ≤ 30 minutes. We tolerated if within a bolus the rate was below the minimal rate for no more than 5 minutes.

**Fig. e2**:


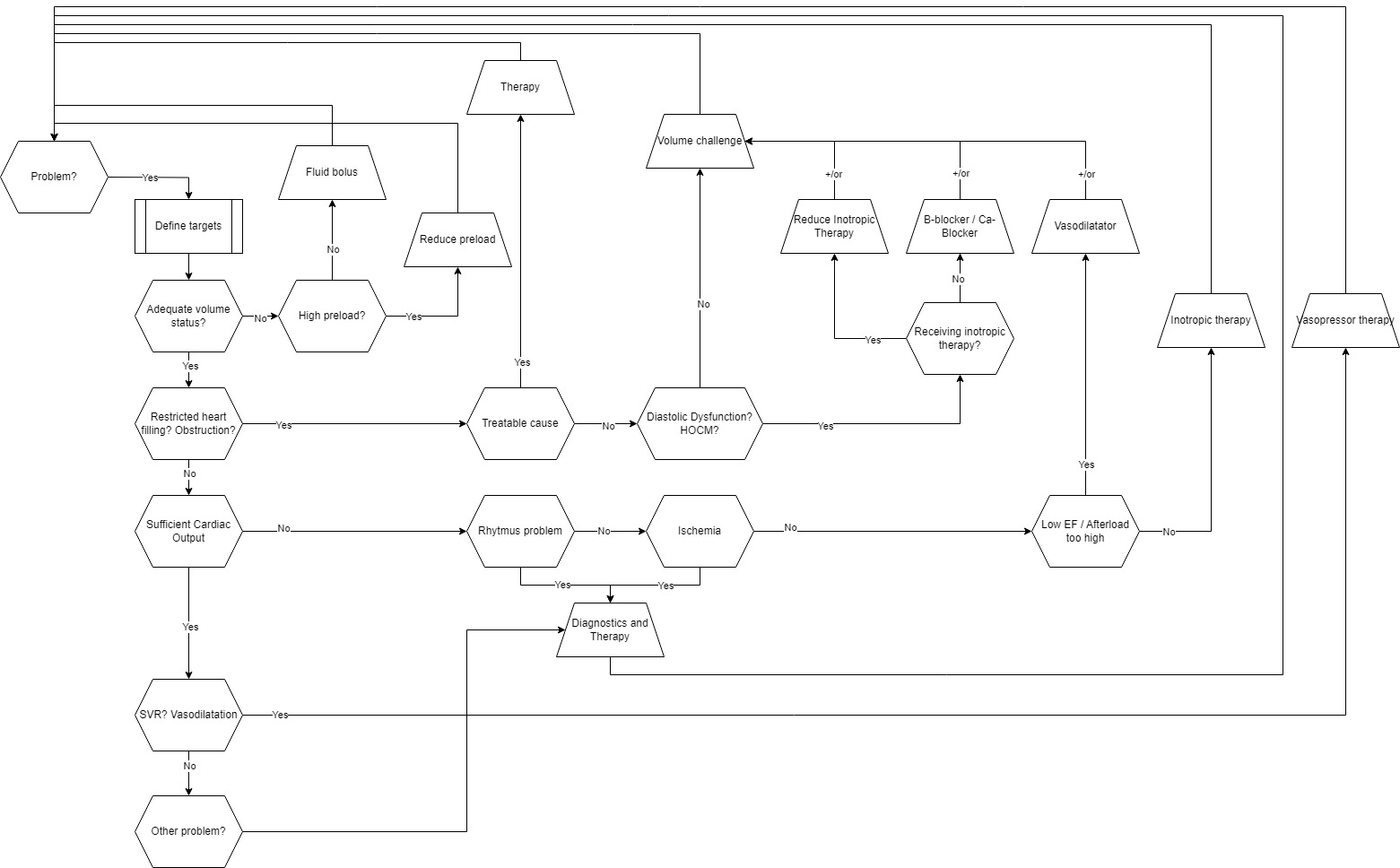


**Fig e2 Translated excerpt of the institution’s hemodynamic protocol.**

SVR: Systemic vascular resistance; EF: Ejection fraction

**Fig. e3**:


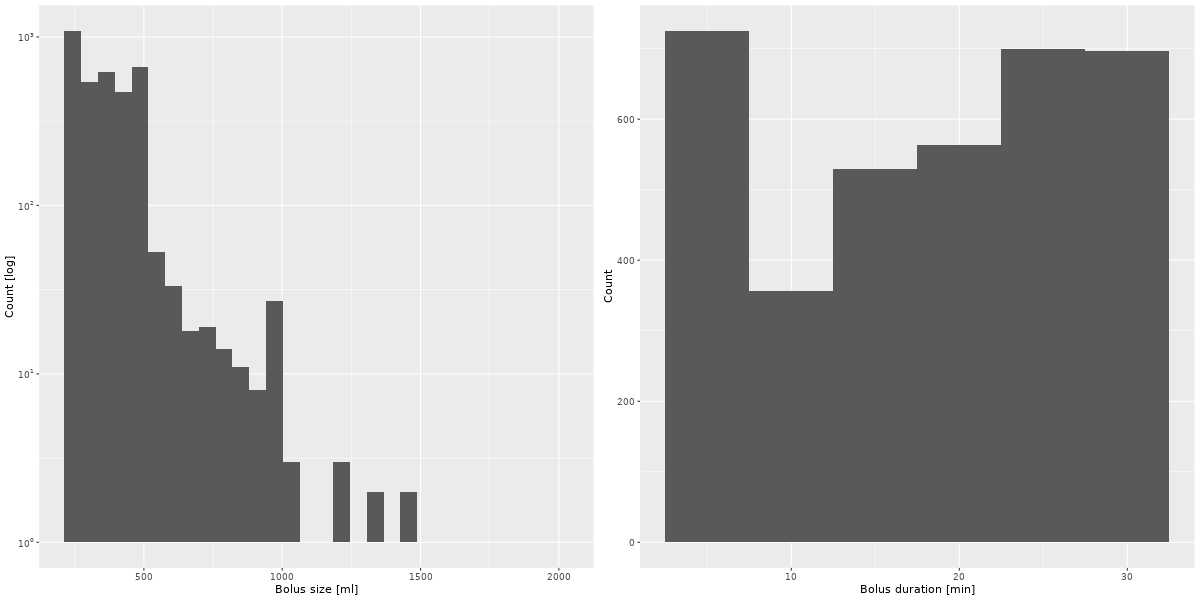


**Fig e3 Bolus size and duration.**

Bolus size refers to the total milliliters (ml) of Ringer’s lactate infused during a bolus episode. Bolus duration is defined as the time in minutes (min) from start to end of the bolus, partitioned into 5min bins.

**Fig. e4**


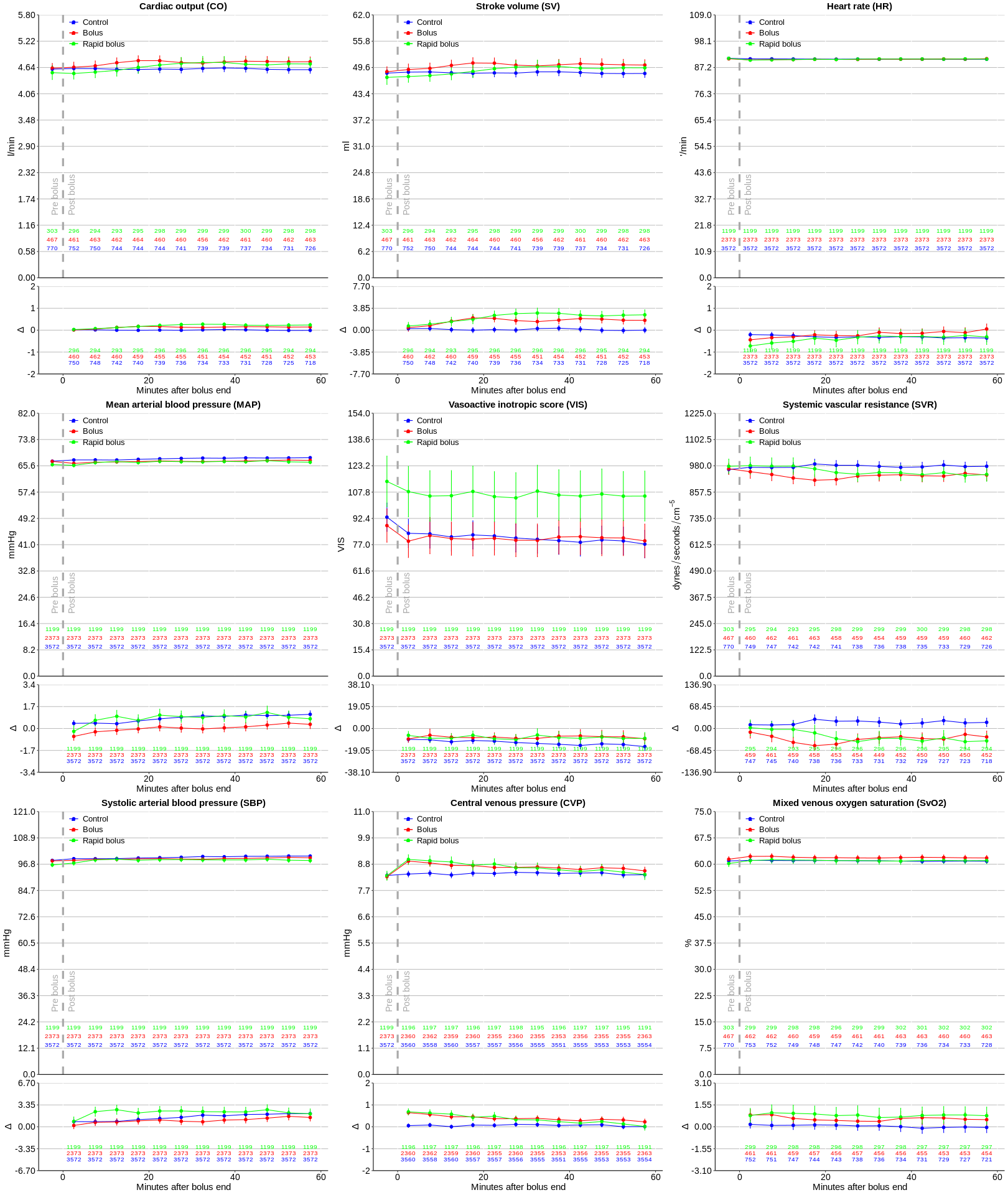


**Fig e4 Hemodynamic effects of FBT for large and rapidly infused FB.**

a-j: for each variable, the top plot shows the average value of 30min before the bolus fluid therapy (before gray line) and the average values of 5minute intervals from 0 to 60minutes after the FB end. In the lower plot the values are the mean of the changes from baseline calculated for each time point individually. In both plots this is shown for the FBT and control episodes separately. The subgroup of FB in the top tercile for fluid bolus rate is displayed separately.

The 95% confidence intervals are shown as error bars. The red and blue numbers represent the number of measurements at each time point for FB (red) and control episodes (blue).

**Fig. e5**


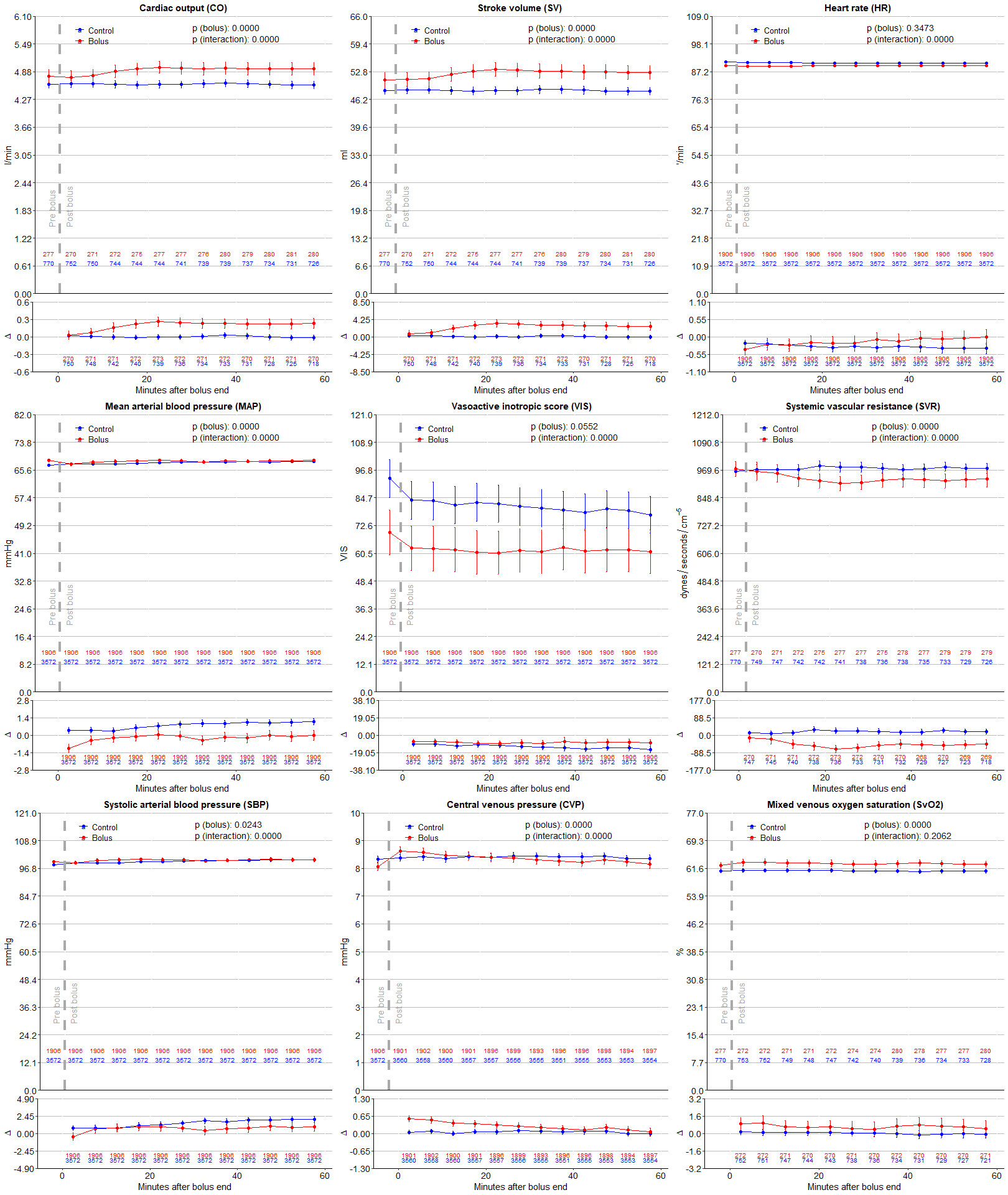


**Sensitivity analysis including the first isolated fluid bolus of the patient only**The controls remain unchanged. a-j: for each variable, the top plot shows the average value of 30min before the bolus fluid therapy (before gray line) and the average values of 5minute intervals from 0 to 60minutes after the FB end. In the lower plot the values are the mean of the changes from baseline calculated for each time point individually. In both plots this is shown for the FBT and control episodes separately. The 95% confidence intervals are shown as error bars. The red and blue numbers represent the number of measurements at each time point for FB (red) and control episodes (blue).

**Fig. e6:**


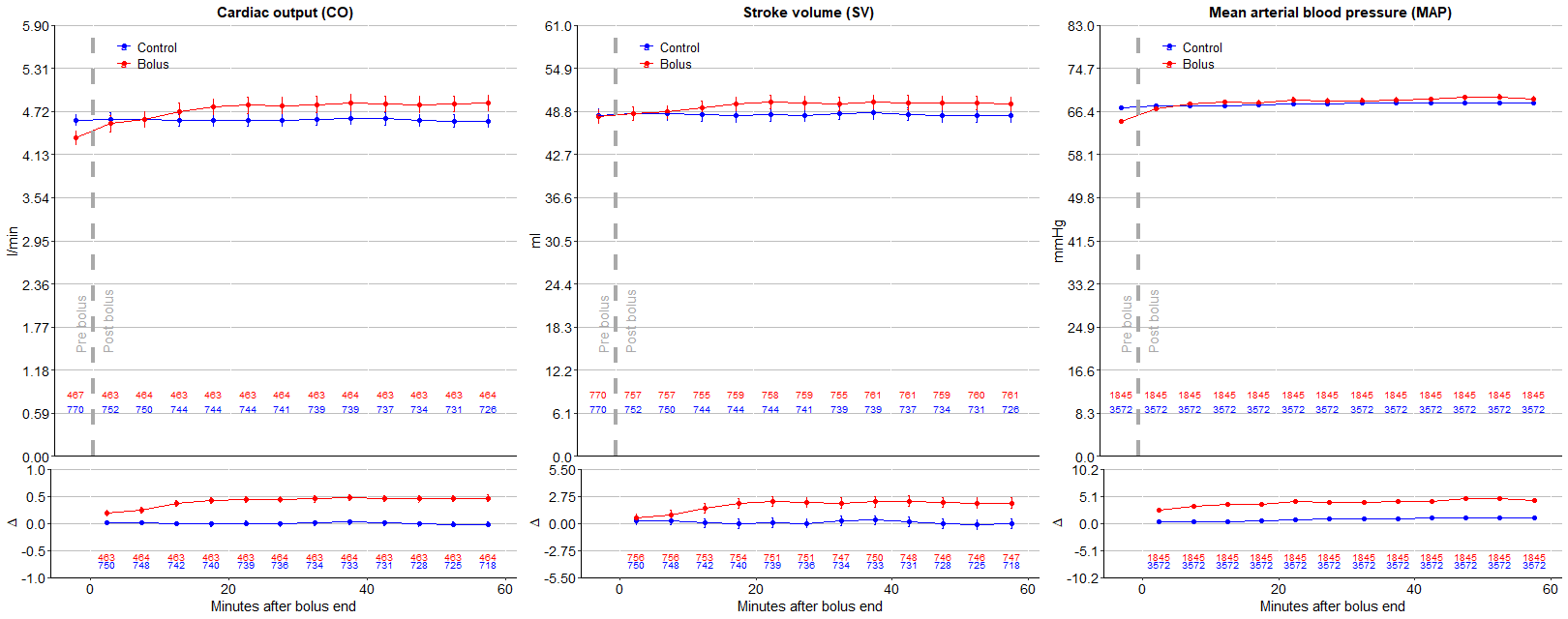


**Fig e6 Change in CO, MAP, SV for episodes with ≥ 10% increase in respective variable compared to baseline.**

The top plot shows the baseline value (mean value of 30min before the bolus fluid therapy, before the gray line) and the mean values of 5-minute intervals from 0 to 60 minutes after the FB end. In the lower plot, the values are the mean of the changes from baseline calculated for each time point individually. In both plots, this is shown for the FBT (red) and control episodes (blue) separately. The 95% confidence intervals are shown as error bars. The red and blue numbers represent the number of measurements at each time point for FB (red) and control episodes (blue).

**Method Supplement:**

**Definition of Baseline and outcome values**

Specific time epochs for baseline (before the FB) and outcome (after FB) values were defined a priori for continuously and sporadically recorded data. For sporadically recorded data (e.g. urine output), we defined the baseline value as the mean of all measurements in the hour before the fluid bolus and the outcome value as the mean of all measurements in the hour after the fluid bolus. For continuously measured data (e.g. stroke volume), we defined the baseline value as the mean of all measured values in the 30 minutes before the fluid bolus. Outcome values were the mean of the measurements for every 5 minute interval in the hour after the end of the fluid bolus. For the purpose of this study, we defined a positive response to FBT as a >10% increase in the variable of interest.

**Technical details of Propensity score matching**

Propensity score matching was performed across FB of all patients. In the PSM model, we included baseline values of heart rate (HR), systolic (SBP) and mean (MAP) arterial blood pressure, central venous pressure (CVP), cardiac output (CO), mean pulmonary arterial pressure (PAPm), pulmonary capillary wedge pressure (PCWP), mixed venous oxygen saturation (SvO2), all vasoactive drugs (Norepinephrine, Epinephrine, Dobutamine, Milrinone, Levosimendan, Theophylline, Vasopressin), urinary output, maintenance fluid administered during the last 30min and the last 2h before FBT, time since ICU admission and mechanical ventilation status. We also included demographic characteristics such as age at admission, sex, Acute Physiology and Chronic Health Evaluation (APACHE) II score and admission type (emergency or elective). Finally, we considered the linear trend (the slope of a linear regression model) in the baseline period for HR and MAP. We followed the recommendations by Blake et al. (1) and obtained separate propensity score models for each pattern of missing values (e.g. separate propensity models for patients with and without pulmonary catheter measurements). The approach appeared valid in our case, as we could reasonably assume that the missing data in the database was indeed not available to the treating physician. Rare combinations of missing values (e.g. only diastolic blood pressure measurement missing) were not considered in our analysis as no reliable propensity score model can be derived for a small subgroup. We, therefore, did not consider patterns with less than 100 observations. Greedy nearest neighbor one-to-one propensity score matching was performed without replacement using a caliper of 0.2. Scores were estimated using a generalized linear model (logistic regression). Matching quality was evaluated by visualizing absolute standardized differences and no hypothesis testing regarding the matching quality was done (2, 3). As recommended, we considered a 10% difference as desirable. The standardized difference was defined as:

$$d=\frac{100 x \left( \bar{x}_{bolus}-\bar{x}_{control} \right)}{\sqrt{\frac{\overline{s}_{bolus}-\overline{s}_{control}}{2}}}$$

Where $\bar{x}_{bolus}, \bar{x}_{control}, \overline{s}_{bolus}, \overline{s}_{control}$are the specific variable’s means and variances in the FB and control episodes.

**References**

1. Blake HA, Leyrat C, Mansfield KE, et al.: Propensity scores using missingness pattern information: a practical guide. *Stat Med* 2020; 39:1641–1657

2. Williamson E, Morley R, Lucas A, et al.: Propensity scores: from naive enthusiasm to intuitive understanding. *Stat Methods Med Res* 2012; 21:273–293

3. Gayat E, Pirracchio R, Resche-Rigon M, et al.: Propensity scores in intensive care and anaesthesiology literature: a systematic review. *Intensive Care Med* 2010; 36:1993–2003
